# Nipah Virus Antibodies in Bats, the Philippines, 2013–2022

**DOI:** 10.3201/eid3108.250210

**Published:** 2025-08

**Authors:** Yoshihiro Kaku, Shumpei Watanabe, Joseph S. Masangkay, Phillip Alviola, Satoshi Taniguchi, Edison Cosico, Yumi Une, Frances C. Recuenco, Satoko Sugimoto, Kentaro Kato, Shigeru Kyuwa, David Emmanuel M. General, Allen John F. Manalad, Sheryl A. Yap, Hironori Bando, Nanako Isobe, Yui Sakata, Shione Takeguchi, Hikaru Fujii, Masayuki Shimojima, Shigeru Morikawa, Ken Maeda, Tsutomu Omatsu

**Affiliations:** National Institute of Infectious Diseases, Japan Institute for Health Security, Tokyo, Japan (Y. Kaku, S. Sugimoto, M. Shimojima, S. Morikawa, K. Maeda); Okayama University of Science, Imabari, Japan (S. Watanabe, Y. Une, N. Isobe, Y. Sakata, S. Takeguchi, H. Fujii, S. Morikawa); College of Veterinary Medicine, University of the Philippines Los Baños, Los Baños, the Philippines (J.S. Masangkay, A.J.F. Manalad); Museum of Natural History, University of the Philippines Los Baños, Los Baños (P. Alviola, E. Cosico, D.E.M. General, S.A. Yap); Graduate School of Medicine, University of Tokyo, Tokyo (S. Taniguchi); College of Science, De La Salle University, Manila, the Philippines (F.C. Recuenco); Graduate School of Agricultural and Life Sciences, University of Tokyo, Tokyo (S. Kyuwa); Tohoku University, Osaki, Japan (K. Kato, H. Bando); Center for Infectious Disease Epidemiology and Prevention Research, Faculty of Agriculture, Tokyo University of Agriculture and Technology, Tokyo (T. Omatsu)

**Keywords:** Nipah virus, viruses, zoonoses, henipavirus, paramyxovirus, bats, neutralizing antibody, the Philippines, meningitis/encephalitis

## Abstract

In 2014, an outbreak of zoonotic Nipah virus (NiV) occurred on Mindanao Island, the Philippines. We investigated the prevalence of NiV in Philippine bats. Because neutralizing antibodies were detected in insectivorous bats on Siargao Island, public health officials should consider that the distribution range of NiV is not limited to Mindanao Island.

Nipah virus (NiV; family *Paramyxoviridae*, genus *Henipavirus*) was first discovered in 1998–1999. Officials in Malaysia and Singapore identified it as a causative virus of severe respiratory disease in pigs and highly fatal encephalitis or respiratory disease in humans (*1*). Subsequently, Bangladesh and India have reported sporadic outbreaks of the virus almost annually (*2*,*3*). Direct bat-to-human transmission is assumed in those outbreaks; however, human-to-human transmission through concentrated contact has also been reported (*3*). 

In Southeast Asia, some frugivorous bat species (mainly of the genus *Pteropus*) and several insectivorous bat species (genera *Hipposideros*, *Scotophilus*, and *Rhinolophus*) are reservoirs of the virus, which has led to its widespread transmission (*4*–*6*). In 2014, in Sultan Kudarat Province, which is located in the southern part of Mindanao Island in the Philippines, 10 horses died, and serious infections occurred in 17 humans, mainly in those who had slaughtered horses or consumed horse meat (*7*). The humans who died had acute encephalitis syndrome, a severe influenza-like illness, or meningitis, and the etiology was diagnosed as henipavirus infection on the basis of neutralizing antibody detection in patient serum samples. One patient had a short 71-bp fragment sequence that was 99% homologous to the NiV strain from Malaysia, suggesting that NiV was the etiologic virus (*7*). The likely source of infection in horses is bats, which are a natural host of the virus.

Residual serum samples used in epidemiologic studies of bat-derived viruses conducted before 2019 were reused in this NiV epidemiologic study (*8*). In addition, we conducted new bat trapping at the end of 2022. In each study, we collected specimens from wild bats.

We attempted to detect NiV-neutralizing antibodies by using serum samples collected from bats in 6 regions of the Philippines, spanning from north to south (Figure). We determined the neutralization titer of each serum sample by using a surrogate assay without an infectious NiV, as previously established (*9*). Using vesicular stomatitis virus expressing secreted alkaline phosphatase pseudotyped with G and F proteins of the NiV strain from Malaysia (VSV-NiV-SEAP) (*9*), we determined the titer of the neutralizing antibody. Moreover, we performed detection of NiV RNA with reverse transcription PCR by using consensus primers that widely detect paramyxoviruses (PAR-F1, PAR-F2, and PAR-R) (Appendix) (*10*).

**Figure:**
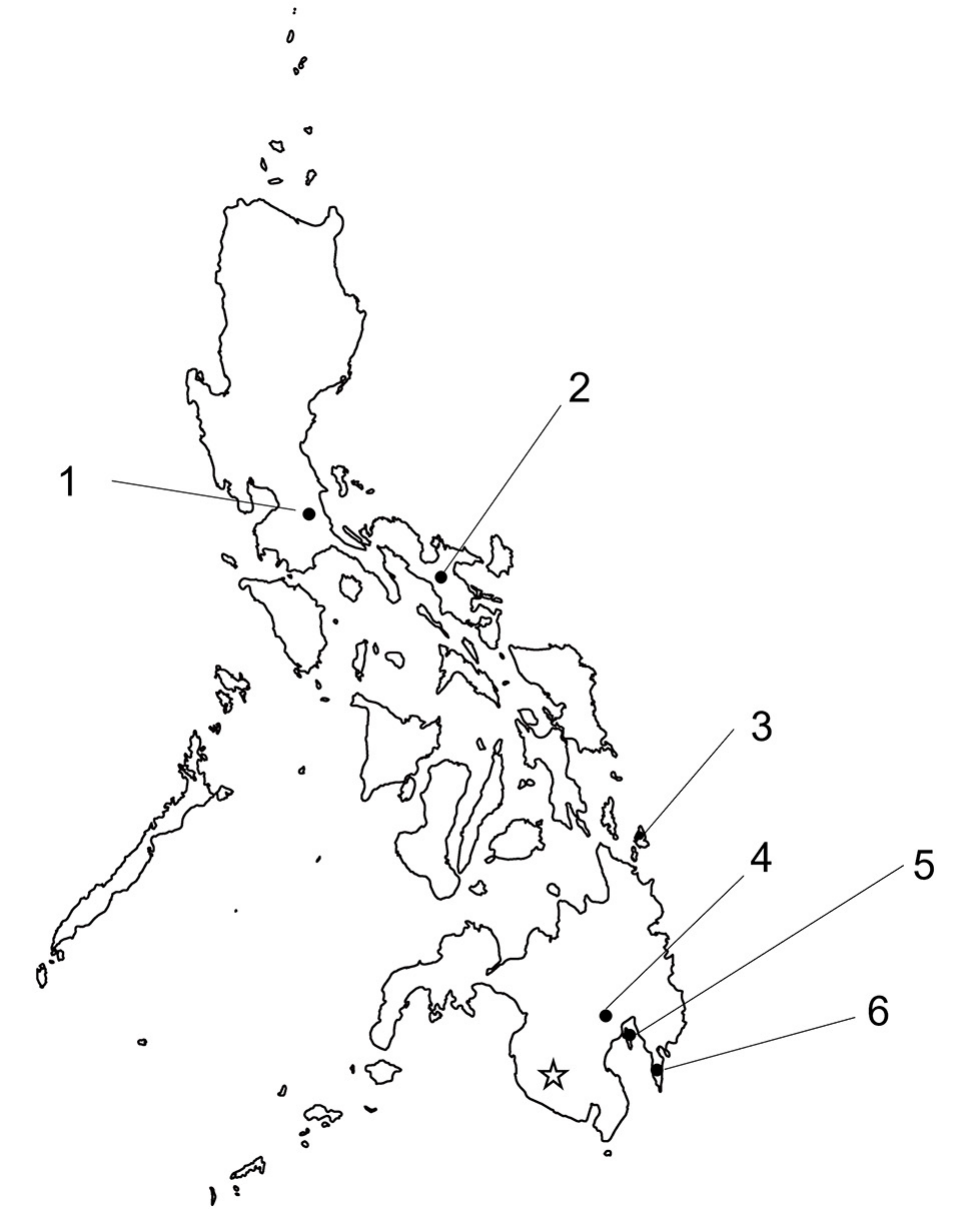
Locations of 6 bat collection sites for Nipah virus antibodies in bats, the Philippines, 2013–2022. 1, U.P. Laguna Quezon Land Grant, Siniloan, Laguna; 2, Naga, Camarines Sur; 3, Siargao Islands, Surigao del Norte; 4, Baguio District, Davao City, Mindanao; 5, Island Garden City of Samal and Talicud Island, Davao del Norte Province; 6, Lavigan, Governor Generoso, Davao Oriental, Mindanao. Star denotes area where Nipah virus outbreaks were reported in 2014.

In total, we diluted 326 bat serum samples 80-fold and screened for VSV-NiV-SEAP (Table) (*9*). We subjected 4 serum samples that tested reactive in screening to serial dilution. We determined antibody titers as values of 16, 41, 47, and 141, which are shown as the reciprocal of the serum dilution factor at which SEAP activity was suppressed by >75% after VSV-NiV-SEAP entered the cells (*9*). We obtained positive samples from the insectivorous bat *Hipposideros diadema*, which was captured on Siargao Island (Figure). We used a similar surrogate system to detect neutralizing antibodies against Hendra virus. The same 4 serum samples showed cell entry inhibition rates ranging from 35.2% to 63.1% against VSV pseudotyped with Hendra virus G and F proteins. Those results were weaker than those obtained for VSV-NiV-SEAP in the screening (Appendix Table). However, because of an insufficient volume of serum samples, we could not perform titration by serial dilution. In contrast, we did not detect any neutralizing antibodies in bats from Mindanao Island or elsewhere (Table). Moreover, we did not detect any viral RNA in reverse transcription PCR targeting paramyxoviruses (including NiV and Hendra virus) using RNA extracted from the 252 samples (collected from serum or spleen) (Table).

**Table:** Neutralizing antibody titers in serum samples from 13 bat species for Nipah virus antibodies in bats, the Philippines, 2013–2022*

Bat species	No. positive/no. tested using pVSV-SNT							No. positive/no. tested using PaV RT-PCR					
	Site 1	Site 2	Site 3	Site 4	Site 5	Site 6		Site 1	Site 2	Site 3	Site 4	Site 5	Site 6
*Cynopterus luzoniensis*	0/41	0/25	0/22	NA	NA	NA		0/28	ND	0/17†	NA	NA	NA
*Eonycteris spelaea*	0/3	NA	0/2	NA	0/13	NA		0/3	NA	0/2†	NA	0/13	NA
*Haplonycteris fischeri*	0/1	NA	NA	NA	NA	NA		ND	NA	NA	NA	NA	NA
*Macroglossus minimus*	0/1	NA	0/9	0/1	NA	NA		ND	NA	0/6†	0/1	NA	NA
*Ptenochirus jagori*	0/63	0/22	0/17	NA	NA	NA		0/63	ND	0/12†	NA	NA	NA
*Rousettus amplexicaudatus*	0/5	NA	0/3	NA	0/44	0/19		0/5	NA	0/1†	NA	0/46	0/20
*Hipposideros coronatus*	NA	NA	ND	NA	NA	NA		NA	NA	0/1†	NA	NA	NA
*Hipposideros diadema*	NA	NA	4/23	NA	NA	NA		NA	NA	0/24†	NA	NA	NA
*Hipposideros obscurus*	NA	NA	ND	NA	NA	NA		NA	NA	0/9†	NA	NA	NA
*Hipposideros pygmaeus*	NA	NA	0/1	NA	NA	NA		NA	NA	ND	NA	NA	NA
*Rhinolophus arcuatus*	NA	NA	0/1	NA	NA	NA		NA	NA	0/1†	NA	NA	NA
*Miniopterus eschscholtzii*	NA	NA	0/3	NA	NA	NA		NA	NA	ND	NA	NA	NA
*Scotophilus kuhlii*	NA	0/7	NA	NA	NA	NA		NA	ND	NA	NA	NA	NA

*Site 1, Laguna 2022; site 2, Naga 2019; site 3, Siargao 2019; site 4, Baguio district 2013 (in Davao City); site 5, Samal and Talicud 2013; site 6, Lavigan 2013 (in Governor Generoso Municipality). NA, not applicable; ND, not done; PaV, paramyxovirus; pVSV-SNT, serum neutralizing test using vesicular stomatitis virus pseudovirus expressing the Nipah virus surface proteins (*9*); RT-PCR, reverse transcription PCR. †RT-PCRs were performed by using RNAs extracted from spleen and not from serum samples.

In this study, we investigated the prevalence of NiV with bat serum samples collected from 6 regions in the Philippines (Figure). We did not detect any antibodies on Mindanao Island, where the henipavirus outbreak occurred, which may be partially because we could not capture and study the primary reservoir, *Pteropus* bats, which fly and migrate at high altitudes. However, we detected NiV antibodies in 4 samples from 1 insectivorous bat species on Siargao Island (Table), which is geographically close, indicating that the distribution range of NiV is not limited to within Mindanao Island.

Antibodies have been reported from other *Hipposideros* bat species closely related to *H. diadema* (*5*). We also captured a species (*Scotophilus kuhlii*) other than *Pteropus* bats, for which antibodies were similarly detected in bats in previous reports (*5*), but we did not detect any antibodies. In contrast, we could not detect viral RNA in all samples because of the small number of samples. We consider it crucial to obtain more viral genetic information to understand the nature of the virus responsible for the henipavirus epidemic in the Philippines and to take countermeasures. More detailed surveys with larger sample sizes on Mindanao Island and surrounding areas are needed. Surveillance of NiV carriage in bats in the Philippines is necessary to characterize the virus, investigate risk factors for future outbreaks of henipavirus, and implement control measures.

Appendix Additional information for Nipah virus antibodies in bats, the Philippines, 2013–2022.
