# Supplementary material for: Nipah Virus Antibodies in Bats, the Philippines, 2013–2022
Source: Emerg Infect Dis. 2025 Aug;31(8):1670–3. doi: 10.3201/eid3108.250210 (PMC12309753; doi:10.3201/eid3108.250210)
Supplement: Appendix — Additional information for Nipah virus antibodies in bats, the Philippines, 2013–2022. [file 25-0210-Techapp-s1.pdf]

Article DOI: https://doi.org/10.3201/eid3108.250210

EID cannot ensure accessibility for supplementary materials supplied by authors. Readers who have difficulty accessing supplementary content should contact the authors for assistance.

Nipah Virus Antibodies in Bats, the Philippines, 2013–2022

Appendix

Supplementary Methods

RT-PCR Using Consensus Primers Targeting Viruses Belonging to the *Paramyxoviridae* Family

RNA was extracted from serum or spleen using a MagMAX Total Nucleic Acid Isolation kit (Thermo Fisher Scientific, Waltham, MA) or TRIzol Reagent (Thermo Fisher Scientific), respectively, according to the manufacturer's protocol. Subsequently cDNA synthesis was performed using SuperScript III RT system (Invitrogen, Waltham, MA, USA) with random primers according to the manufacturer's protocol. Semi-nested PCRs were then performed using GoTaq DNA polymerase (Promega, Madison, WI, USA) in accordance with the prior report (*1*).

Reference

1. Tong S, Chern SW, Li Y, Pallansch MA, Anderson LJ. Sensitive and broadly reactive reverse transcription-PCR assays to detect novel paramyxoviruses. J Clin Microbiol. 2008;46:2652–8. PubMed https://doi.org/10.1128/JCM.00192-08

Appendix Table. Observed percentage inhibition rate of cell entry with pseudo-typed VSVs*

Bat ID	Neutralization titers determined by VSV expressing NiV G and F	Percent inhibition rate of the VSV expressing NiV G and F at 1:80 serum dilution	Percent inhibition rate of the VSV expressing HeV G and F at 1:80 serum dilution
Siargao4147	16	53.3	35.2
Siargao4156	41	70.1	52.6
Siargao4131	47	69.9	50.9
Siargao4135	141	78.3	63.1

*HeV, hendra virus; NiV, nipah virus; VSV, vesicular stomatitis virus.
